# Evaluating the resistance mechanism of *Atriplex leucoclada* (Orache) to salt and water stress; A potential crop for biosaline agriculture

**DOI:** 10.3389/fpls.2022.948736

**Published:** 2022-08-01

**Authors:** Hasnain Alam, Muhammad Zamin, Muhammad Adnan, Nisar Ahmad, Taufiq Nawaz, Shah Saud, Abdul Basir, Ke Liu, Matthew Tom Harrison, Shah Hassan, Hesham F. Alharby, Yahya M. Alzahrani, Sameera A. Alghamdi, Ali Majrashi, Basmah M. Alharbi, Nadiyah M. Alabdallah, Shah Fahad

**Affiliations:** ^1^Department of Biological Sciences, International Islamic University, Islamabad, Pakistan; ^2^Department of Biology, College of Sciences, United Arab Emirates University, Al Ain, United Arab Emirates; ^3^Department of Agriculture, University of Swabi, Swabi, Pakistan; ^4^Department of Biotechnology, University of Science and Technology, Bannu, Pakistan; ^5^Department of Food Science and Technology, The University of Agriculture, Peshawar, Pakistan; ^6^College of Life Science, Linyi University, Linyi, China; ^7^Tasmanian Institute of Agriculture, University of Tasmania, Burnie, TAS, Australia; ^8^Department of Agricultural Extension Education & Communication, The University of Agriculture, Peshawar, Pakistan; ^9^Department of Biological Sciences, Faculty of Science, King Abdulaziz University, Jeddah, Saudi Arabia; ^10^Department of Biology, College of Science, Taif University, Taif, Saudi Arabia; ^11^Department of Biology,, Faculty of Science, University of Tabuk, Tabuk, Saudi Arabia; ^12^Department of Biology, College of Science, Imam Abdulrahman Bin Faisal University, Dammam, Saudi Arabia; ^13^Hainan Key Laboratory for Sustainable Utilization of Tropical Bioresource, College of Tropical Crops, Hainan University, Haikou, China; ^14^Department of Agronomy, The University of Haripur, Haripur, Pakistan

**Keywords:** salt stress, water stress, *A. leucoclada*, alternate crops, saline agriculture

## Abstract

The development of food and forage crops that flourish under saline conditions may be a prospective avenue for mitigating the impacts of climate change, both allowing biomass production under conditions of water-deficit and potentially expanding land-use to hitherto non-arable zones. Here, we examine responses of the native halophytic shrub Atriplex leucoclada to salt and drought stress using a factorial design, with four levels of salinity and four drought intensities under the arid conditions. A. leucoclada plants exhibited morphological and physiological adaptation to salt and water stress which had little effect on survival or growth. Under low salinity stress, water stress decreased the root length of A. leucoclada; in contrast, under highly saline conditions root length increased. Plant tissue total nitrogen, phosphorus and potassium content decreased with increasing water stress under low salinity. As salt stress increased, detrimental effects of water deficit diminished. We found that both salt and water stress had increased Na^+^ and Cl^–^ uptake, with both stresses having an additive and beneficial role in increasing ABA and proline content. We conclude that A. leucoclada accumulates high salt concentrations in its cellular vacuoles as a salinity resistance mechanism; this salt accumulation then becomes conducive to mitigation of water stress. Application of these mechanisms to other crops may improve tolerance and producitivity under salt and water stress, potentially improving food security.

## Introduction

Climate change has increased the global mean temperature and water scarcity throughout the world (Zamin et al., 2019a). Demand for water supply is continuously increasing and had increased threefold since the 1950s as the freshwater supply has been on the wane (Gleick, 2003; Wu and Tan, 2012). According to an estimate, an average of 90% of global fresh water is being used for agriculture (Shiklomanov, 2000). According to the (FAO, 2005) report salinity had affected 800 Mha of land globally. It is expected that salinity will annually destroy about 10 Mha of agricultural land (Khan et al., 2006; Zamin et al., 2019b). Thus, the landscaping sector will face serious challenges, to meet irrigation requirements (Shahin and Salem, 2014a).

To solve the problems of water shortage and groundwater salinity many irrigation systems and much equipment is being introduced (Shahin and Salem, 2014b; Zamin and Khattak, 2018). Two types of approaches had been taken on so far to overcome these problems. The first is modifying the environment by managing the irrigation and drainage and the second approach is genetically modifying the plants to enhance their stress tolerance (Läuchli and Lüttge, 2002; Mahmood et al., 2003). However, stress tolerance responses of plants are complex and the functions of many genes controlling these mechanisms are unknown (Chaves et al., 2003). Halophytes had been recommended as a solution for production with salt/brackish water (Khan and Weber, 2006). Native halophyte plants can grow in harsh environmental conditions and can be introduced into urban landscaping under drought and saline conditions (Franco et al., 2006; Zamin and Khattak, 2017).

Increasing water shortage and irrigation water salinity are the main abiotic stresses for the plants. These stresses disturb plant physiology and growth by disrupting their gene expression (Wu and Tan, 2012; Zamin et al., 2018). Plants have evolved numerous mechanisms to adapt to salt stress conditions (Munns and Tester, 2008). Water stress-avoiding refers to a range of morphological and physiological adaptations of plants to sustain suitable water status. Another approach to withstand water stress is water stress tolerance which includes physiological and biochemical mechanisms (Clarke and Durley, 1981).

*Atriplex leucoclada* (English name: Orache; Arabic name: Ragal, رغل), a halophytic plant, is a low perennial shrub that has developed various strategies to adapt to saline environments with excessively high salt content in the soil. Originating in the Mediterranean basin, *A. leucoclada* grows in many different habitats, but it usually occurs on sabkha, coastal and inland salt marshes with a high accumulation of salts, and occasionally on silty soils. *A. leucoclada* is an important species for agricultural use in arid regions. Atriplex species can be planted for soil desalination and CO_2_ sequestration. It is a useful plant for desert and extensive landscape schemes as a groundcover, occasionally requiring watering and maintenance to improve its appearance (Arriyadh, 2014).

Studying *A. leucoclada* under stress conditions will help us to better understand the salt and water stress resistance mechanisms. Moreover, introducing the identified species in landscaping will not only save a huge amount of water but also preserve the biodiversity, wildlife habitats, horticulture heritage, and national unique landscape of the country.

## Materials and methods

### Research site

The field experiment was conducted at AL-Foa Research Farm, United Arab Emirates University, Al Ain, Abu Dhabi, United Arab Emirates (24°12′ N and 55° 44′ E) during December 2015–July 2016. The experimental site is situated in the arid region, having a long, hot summer season of 4 months, i.e., from May to September with maximum temperatures above 45°C. The winter prevails from mid-November to the end of February followed by a short spring season from March to April. The mean annual temperature varies between 12 and 45°C during the winter and summer seasons, respectively (SCAD, 2015). Soil type was classified as Typic Torriorthent sandy-skeletal hyperthermic soil and is silty loam having pH (H_2_O) of 8.6, ECe of 8.2 dS m^–1^ with SAR of 22.4 and 0.6% organic matter (Abdelfattah et al., 2009).

### Experimental design

*Atriplex leucoclada* seeds were sown in germinating trays with growing media of potting soil and sweet sand (Red desert sand with low salinity used for agriculture) 1:1 by volume. The soil used in potting mix was sandy in nature having 24.53 percent carbonate content with pH of 7.58 and Ec 9.49. The Ca content of soil sample was 25 mg/kg whereas Mg was 34.2 mg/kg and low K content, i.e., 7.53 mg/kg (Table 1). After 3 weeks of germination, seedlings were transplanted to 20 cm pots filled with red desert sand. Seedlings were thinned to one seedling per pot. The fertilization and weeding practices were equally applied to all treatments during the entire growing period of the plants. After 2 months of growth, four saltwater treatments, i.e., 5 dS m^–1^ (Control; S1), 10 dS m^–1^ (low salinity level; S2), 15 dS m^–1^ (moderate salinity level; S3), and 20 dS m^–1^ (high salinity level; S4) were prepared by dissolving NaCl to fresh water, supplied by Al-Ain municipality (Al-Dakheel et al., 2015; Zamin et al., 2019a). Salinity treatments were prepared in four different water tanks. These water tanks were connected to a drip irrigation line to supply water to each pot individually with four irrigation intestines. To estimate field capacity the fully water-saturated soil was weighed and then dried to constant weight at 105°C. The weight difference between water-saturated and oven-dried soil was taken as the weight of water needed to bring soil to field capacity, and lower FC was calculated accordingly. Four irrigation intensities were: 100% field capacity (Control; WL1), 80% field capacity (low stress; WL2), 60% field capacity (moderate stress; WL3), and 40% field capacity (severe stress; WL4). Irrigation water was quantified for each water stress treatment and was applied to the plants through a drip irrigation system. The experiment was conducted in an open field and plants were grown under natural environmental conditions. The experiment was conducted in a randomized complete block design with a split-plot arrangement replicated three times. The salinity levels were allotted to the main plot while the irrigation intensities were allotted to sub-plots.

**TABLE 1:** Physicochemical properties.

Soil properties	
**Texture**	
Sand (%)	87.5
Silt (%)	5
Clay (%)	7.5
Total carbonate (%)	24.53
EC (dSm^–1^)	9.49
pH	7.58
**Cations (mq L^–1^)**	
Ca^+^	25
Mg	34.2
Na^+^	53.8
K	7.52
**Anions (mq L^–1^)**	
Cl	46.8
HCO_3_	20.4
SO_4_	0.64
Mg:Ca Ratio	1.37

### Harvesting and sampling

Three plants from each treatment were harvested at the end of each month. Data was recorded for morphological parameters each month. For the quantitative chemical analysis, representative specimens of each plant were instantly ground in liquid nitrogen and stored at −80°C.

### Morphological traits

After harvest, the plant samples were carefully cleaned from sands, washed with distilled water, and dried with the help of tissue paper. After harvesting each plant was divided into shoots and roots and oven-dried (60°C) and weighted (± 0.0001 g). For morphological traits, all the samples were put in Ziploc bags, placed in an ice bag at 4°C, and transferred to the laboratory. Shoot length was measured from the base of the stem to the apex end while root length was measured from the root base up to the end of the primary root.

### Physiological traits

Photosynthetic rate (P) of upper, lower, and basal leaves was measured weekly using a Plant Photosynthesis Meter (EARS, Netherlands) (Samarah, 2005). Leaf water potential was recorded during midday using a WP4C Dewpoint psychrometer (Decagon Devices, Inc., United States) (Xiong et al., 2014). Leaf water potential was recorded after 1 and 5 months of treatment application. Phosphorus concentrations were estimated in plant leaves at the end of the experiment by the methodology laid out by Olsen (1954). K and Na^+^ content of plant extracts were determined by Flame Emission Spectroscopy at the end of the experiment. For Cl^–^ content 50 mg of leaf and root samples were ground and heated in distilled water for 3 h (80°C). The Cl^–^ content of the extract was then determined with the chloride analyzer at the end of the experiment.

### Biochemical traits

ABA and Proline extraction was performed on 10 mg of freeze-dried leaf tissue as described by Forcat et al. (2008). The samples were analyzed for ABA and Proline using LCMS/MS and were filtered through a 0.45 μm cellulose acetate syringe. The phytohormones separation was done using a C18 column (ZORBAX Eclipse Plus). An injection of 2 μl was loaded onto the C18 column (1.8 μm particle size, 2.1 mm inner diameter and 50 mm long) at a flow rate of 0.2 mL/min and the column temperature was kept at 35°C. The liquid chromatography was connected to an Agilent Technologies Mass Spectrometry (6420 Triple Quad detector). For elution solvent A consist of formic acid (0.1%) with distilled water and solvent B consisted of an LCMS grade acetonitrile was used. The analytical procedure was as follows: Solvent A was used (5 min), then the gradient from 0 to 100% solvent B was used, (5–20 min) after the solvent B was kept constant (5 min) and at 25.1 min solvent A was 100% was used for (30-min). During the analysis with LC-MSMS only negative polarity mode was used for ABA and Proline analysis. For fragmentation nitrogen gas was used. The capillary voltage was 4,000 V, the gas flow was 8 L/min, the gas temperature was 300°C and the nebulizer pressure was 45 psi.

### Statistical analysis

Two-way (ANOVA) was used to check the effect of salinity and drought, and their interaction on morphological, biochemical, and physiological traits. The normality was checked with the Shapiro–Wilk test. The *post-hoc* Tukey’s HSD was used to check the comparison between treatments. All the analysis was performed using the SPSS software.

## Results and discussion

### Percent survival (%), root and shoot length (cm), and weight (g)

Results concerning the morphological response of *A. leucoclada* to varying salt and drought stresses are given in Table 2. Analysis of variance revealed that all the morphological parameters (percent survival, root and shoot length, and weight) did not show any significant (*P* > 0.05) effect from salt stress. It is clear from the results of survival percentage that *A. leucoclada* can survive on all studied salt and water stress levels. Analysis of variance revealed that different water stress levels significantly (*P* ≤ 0.05) affected Shoot dry weight (SDW), RDW, and SL of *A. leucoclada*. Increasing the water stress level decreased the SDW. The highest SDW was recorded for WL1 (18.15 g) while the other three water stress levels have lower SDW. Maximum RDW was measured for WL1 (3.72 g) while the other three water levels of WL2, WL3, and WL4 were significantly lower and like each other. Maximum SL was recorded for 44.37 cm for WL1 while other three water levels of WL2, WL3, and WL4 were significantly lower and similar to each other. Different salt and water stress levels and interaction of salt and water stress levels had a non-significant effect (*P* > 0.05) on the RL of *A. leucoclada*.

**TABLE 2:** Morphological response of *Atriplex leucoclada* to varying drought and salinity stresses.

Drought stress (% field capacity)	Survival percentage	Root length (cm)	Shoot length (cm)	Root dry weight (g)	Shoot dry weight (g)
100 (WL1)	87.82	40.12	44.37	3.72	18.15
80 (WL2)	77.46	33.56	23.83	1.84	7.43
60 (WL3)	80.96	34.26	20.56	1.62	4.68
40 (WL4)	81.02	31.68	21.22	1.70	6.34
LSD (0.05)	NS	NS	14.449	1.2453	6.2891
**Salinity stress (dS m^–1^)**					
5 (S1)	88.48	37.89	27.05	1.92	7.70
10 (S1)	77.65	36.77	34.64	2.08	9.56
15 (S1)	77.81	33.26	30.07	2.37	10.61
20 (S1)	82.64	31.70	18.22	2.50	8.74
LSD	NS	NS	NS	NS	NS
**Interaction (WS × SS)**	**NS**	**NS**	**NS**	**NS**	**NS**

Means with different letters in each category are significantly different at α = 0.05. NS, WS, SS, and LSD stand for non-significant, drought stress, salinity stress, and least significant difference, respectively.

### Photosynthetic rate (Pr) (μmol/m^2^/s), leaf water potential (MPa), Na^+1^ uptake (μ mole g^–1^), Cl^–1^ uptake (μ mole g^–1^)

Different salt and water stress levels had a significant (*P* ≤ 0.05) effect on the Pr of *A. leucoclada* (Table 3). Pr increased with increasing water stress while decreasing with increasing salt stress.

**TABLE 3:** Physiological response of ***Atriplex leucoclada*** to varying drought and salinity stresses.

Drought stress (% field capacity)	Photosynthetic rate (μmol m^–2^ S^–1^)	Leaf water potential (MPa)	Na^+1^ uptake (μ mole g^–1^)	Cl ^–1^ uptake (μ mole g^–1^)
100 (WL1)	15.4a	−47	482	278
80 (WL2)	18.3a	−54	475	229
60 (WL3)	29.0b	−55	489	179
40 (WL4)	24.9b	−52	500	255
LSD (0.05)	10.216	2.6121	13.719	45.431
**Salinity stress (dS m^–1^)**				
5 (S1)	23.9c	−55	440	104
10 (S1)	25.9bc	−48	493	199
15 (S1)	19.0a	−56	496	287
20 (S1)	18.8ab	−50	518	352
LSD	4.5867	3.6015	17.832	49.458
**Interaction (WS × SS)**	**NS**	**Figure 1**	**Figure 2**	**Figure 3**

Means with different letters in each category are significantly different at α = 0.05. NS, WS, SS, and LSD stand for non-significant, drought stress, salinity stress, and least significant difference, respectively.

**FIGURE 1:**
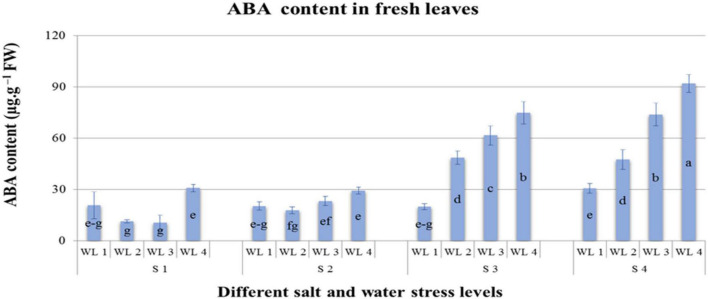
Interactive effect of salinity and drought stress on ABA content (μg. g^–1^ FW) content of *Atriplex leucoclada*. Error bars represent the standard error of the mean (*n* = 3). WL stands for water level (WL1 = 100% field capacity, WL2 = 80% field capacity, WL3 = 60% field capacity and WL4 = 40% field capacity) while S represents salinity (S1 = 5 dS m^–1^, S2 = 10 dS m^–1^, S3 = 15 dS m^–1^, and S4 = 20 dS m^–1^). Letters a–g indicate whether the bar graphs showing parameters are significant or nonsignificant. Bars having the same letters are nonsignificant.

**FIGURE 2:**
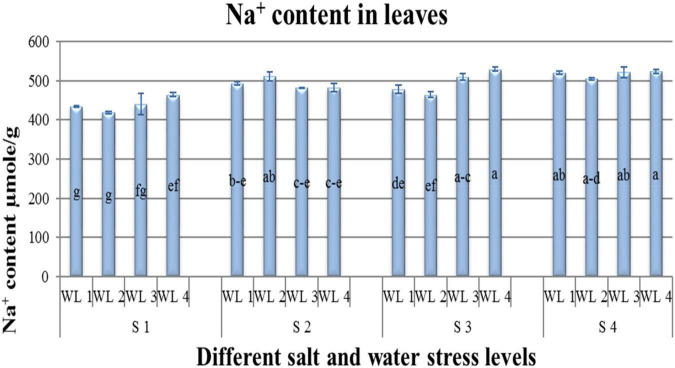
Interactive effect of salinity and drought stress on Na^+^ content of *Atriplex leucoclada*. The error bars represent the standard error of the mean (*n* = 3). WL stands for water level (WL1 = 100% field capacity, WL2 = 80% field capacity, WL3 = 60% field capacity and WL4 = 40% field capacity) while S represents salinity (S1 = 5 dS m^–1^, S2 = 10 dS m^–1^, S3 = 15 dS m^–1^, and S4 = 20 dS m^–1^). Letters a–g indicate whether the bar graphs showing parameters are significant or nonsignificant. Bars having the same letters are nonsignificant.

**FIGURE 3:**
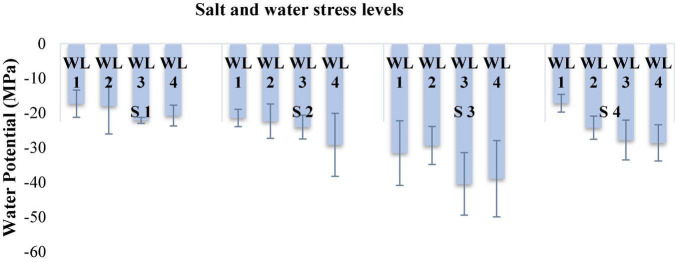
Interactive effect of salinity and drought stress on water potential (MPa) of *Atriplex leucoclada*. The error bars represent the standard error of the mean (*n* = 3). WL stands for water level (WL1 = 100% field capacity, WL2 = 80% field capacity, WL3 = 60% field capacity and WL4 = 40% field capacity) while S represents salinity (S1 = 5 dS m^–1^, S2 = 10 dS m^–1^, S3 = 15 dS m^–1^, and S4 = 20 dS m^–1^).

Salt × water stress had a significant effect (*P* ≤ 0.05) on the leaf water potential of *A. leucoclada*. After months of treatment application, S2WL1 had a maximum LWP of −46 MPa, and a minimum LWP (−64 MPa) was recorded for S1WL2 Table 3 and Figure 1.

Na^+^ and Cl^–^ content had an interactive effect (*P* ≤ 0.05) on salt and water stress. Na^+^ and Cl^–^ content increased not only with increasing salt stress but also with increasing water stress. Na^+^ was lower in S1WL2 and S1WL1 and increased with increasing salinity and water stress. Maximum Na^+^ content was reported for S3WL4 and S4WL4 (Figure 2). Similarly, the lowest Cl^–^ content was recorded for S1WL1 and S1WL2. The highest Cl^–^ content was recorded for S4WL1 (Table 3 and Figure 3).

### Abscisic acid (μg. g^–1^ FW) and proline content (μg. g^–1^ FW)

**FIGURE 4:**
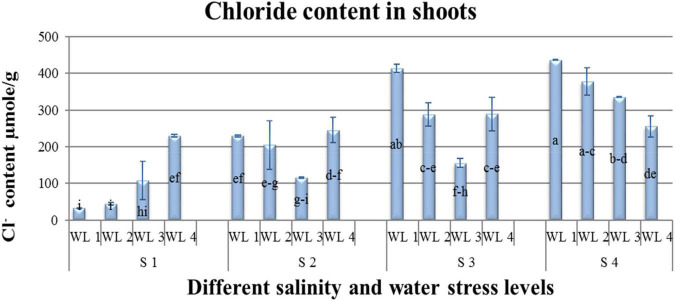
Interactive effect of salinity and drought stress on Cl^–^ content of *Atriplex leucoclada*. Error bars represent the standard error of the mean (*n* = 3). WL stands for water level (WL1 = 100% field capacity, WL2 = 80% field capacity, WL3 = 60% field capacity and WL4 = 40% field capacity) while S represents salinity (S1 = 5 dS m^–1^, S2 = 10 dS m^–1^, S3 = 15 dS m^–1^, and S4 = 20 dS m^–1^). Letters a-i indicate whether the bar graphs showing parameters are significant or nonsignificant. Bars having the same letters are nonsignificant.

**FIGURE 5:**
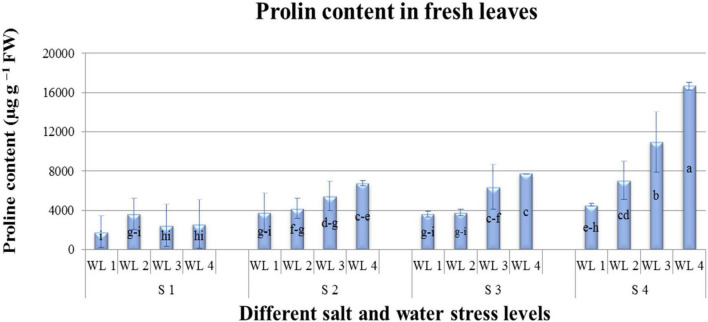
Interactive effect of salinity and drought stress on Proline content (μgg^–1^FW) content of *Atriplex leucoclada*. Error bars represent standard error of the mean (*n* = 3). WL stands for water level (WL1 = 100% field capacity, WL2 = 80% field capacity, WL3 = 60% field capacity and WL4 = 40% field capacity) while S represents salinity (S1 = 5 dS m^–1^, S2 = 10 dS m^–1^, S3 = 15 dS m^–1^, and S4 = 20 dS m^–1^. Letters a–i indicate whether the bar graphs showing parameters are significant or nonsignificant. Bars having the same letters are nonsignificant.

Means of ABA quantified are represented in Table 4 and Figure 4. Salt and water stress levels had statistically significant (*P* ≤ 0.05) interaction for ABA production. ABA production showed an increasing trend with increasing both salt and water stress levels. Maximum ABA content was 92 μg. g^–1^ FW quantified at S4WL4. ANOVA revealed that different salt and water stress levels and their interaction had a significant effect on the proline content of *A. leucoclada*. Proline content increased with increasing the combined effect of salt and water stress and maximum proline content was 16,654 μg^–1^ FW recorded for S4WL4 (Table 4 and Figure 5).

**TABLE 4:** Biochemical response of *Atriplex leucoclada* to varying drought and salinity stresses.

Drought stress (% field capacity)	ABA (μg g^–1^ FW)	Proline (μg g^–1^ FW)
100 (WL1)	23.02	3472.09
80 (WL2)	31.40	4701.77
60 (WL3)	42.38	6355.27
40 (WL4)	56.79	8461.69
LSD (0.05)	5.3	974.17
**Salinity stress (dS m^–1^)**		
5 (S1)	18.46	2668.84
10 (S1)	22.73	5077.64
15 (S1)	51.33	5416.19
20 (S1)	61.07	9828.14
LSD	7.05	1430.2
**Interaction (WS × SS)**	**Figure 4**	**Figure 5**

Means with different letters in each category are significantly different at α = 0.05. NS, WS, SS, and LSD stand for non-significant, drought stress, salinity stress, and least significant difference, respectively.

## Discussion

### Percent survival (%), root and shoot length (cm), and weight (g)

Salt and water stress were mostly studied separately for their effects on crop growth (Hamed et al., 2013). Hence only a few studies had examined their interactions (Hamed et al., 2013). *A. leucoclada* has been found to be resistant to salt and water stress and survived under all salt and water stresses applied. Salt stress had no significant effect on the growth of *A. leucoclada*. On the other hand, increasing water stress had shown a major decrease in growth parameters except for root length (Table 2). Similar results for reduced growth under salt stress as compared to water stress are also resorted by Hassine and Lutts (2010) for *Atriplex halimus*, Melo et al. (2016) for *Atriplex nummularia* and Almas et al. (2013) in *Artemisia vulgaris* and Álvarez et al. (2018) for *Pistacia lentiscus*. The same is reported for *A. canescens* (Glenn and Brown, 1998), *A. lentiformis* (Meinzer and Zhu, 1999), *A. halimus* (Alla et al., 2011), and *Anethum graveolens* (Tsamaidi et al., 2017). Result similar to our experiment for decreased RDW under water stress as compared to salt stress are reported by Wang et al. (2011) for tamarisk (*Tamarix chinensis* Lour), Yagmur and Kaydan (2008) for triticale (*Triticosecale* Witm., cv. Presto), Khan et al. (2017) for soybean and, Miranda-Apodaca et al. (2018) for Quinoa (*Chenopodium quinoa*).

Although soil water potential is decreased by saline water irrigation water flow to the roots remains the same. On the other hand, water stress decreases the soil matric potential and decreases water flow to the roots (Homaee et al., 2002). This can be the reason that the matric potential during water stress affected the shoot growth of studied species more than that did the osmotic potential (Shainberg and Shalhevet, 2012). These results corroborate with the results of Maggio et al. (2005) who reported 22% less aboveground dry weight in salt stress and 46% less dry weight in water stress than control.

### Photosynthetic rate (μmol/m^2^/s), leaf water potential (MPa)

Both salt and water stress had an interactive effect (*P* ≤ 0.05) on Pr of *A. leucoclada*. Pr was maximum during the cooler month of March and start decreasing with increasing temperature in the months after that. Pr was significantly affected by salt x water stress. Salt stress decreased the Pr while water stress increased the Pr significantly. The current results are in line with Wang et al. (2011) for *Tamarix chinensis*. Water deficit led to earlier peaks of net photosynthetic rate (PN) during the day. In the case of quinoa, the highest salt concentration of (500 mM) decreased net Pr by 65% compared to controls. However, water stress resulted in 77% lower values for net Pr (Miranda-Apodaca et al., 2018).

Under water stress, leaf water potential and thus photosynthetic activity is decreased (Razzaghi et al., 2011). This reduction in photosynthesis can be caused by stomatal closure (Goldstein et al., 1996), disturbance of photosynthetic activity (Drew et al., 1990), or at both low and high salt concentrations (Yeo et al., 1991). Decreasing soil moisture content and water potential reduces the water potential of the plant tissue. In response to low water potential additional solutes are accumulated which is referred to as osmotic adjustment (OA) (Zhang et al., 1999; Verslues et al., 2006). In halophyte species, Na^+^ is involved in OA. It is supposed that Na^+^ largely exists in the vacuoles (Martínez et al., 2005).

Salt and water stress had an additive effect on LWP and leaf osmotic potential reduction of *Zygophyllum xanthoxylum* (Ma et al., 2012). Miranda-Apodaca et al. (2018) compared the effect of osmotic and ionic stress on quinoa. It was reported that plants subjected to saline treatment observed a greater capacity for osmotic adjustment. In contrast, plants subjected to water stress treatment showed more dehydration. The water potential diminished significantly due to salt and water stress (González et al., 2012). Álvarez et al. (2018) reported decreased water potential for all salt and water stress treatments in *Pistacia lentiscus*.

It was concluded that osmotic adjustment through the uptake of readily available inorganic ions (Na^+^ and Cl^–^) under salt stress is more efficient than adjustment through the production of organic solutes under water stress (Liu et al., 2008; Slama et al., 2008; Sucre and Suarez, 2011; Álvarez et al., 2012).

Herralde et al. (1998) submitted plants of *Argyranthemum coronopifolium* to salt and water stress independently. Water stress promoted significant differences in leaf water potential (−1.76 MPa for Ψw) in stressed plants vs. control.

Hassine et al. (2008) exposed *Atriplex halimus* plants to 40/160 mM NaCl or 15% polyethylene glycol. Shoot water potential in plants exposed to PEG remained lower than the plants under the highest salt stress. Duarte and Souza (2016) investigated water potentials in *Capsicum annuum* by irrigating with different levels of saline water. Salt stress resulted in a decrease in LWP. The decrease in the osmotic potential in plant leaves was a mean of saline stress adoption.

Our results are in agreement with the findings of Omami and Hammes (2006) in amaranth under salt and water stress, Mannan et al. (2013) for soybean, and Khan et al. (2015) for mung bean under salinity stress. It can be concluded that salt stress can help to reduce the negative effects of water stress by osmotic adjustment through Na^+^ and proline accumulation.

### Na^+1^ uptake (μ mole g^–1^), Cl^–1^ uptake (μ mole g^–1^)

Under saline conditions, Na^+^ in the growth medium might compete with K absorption by the roots (Blumwald, 2000). It is assumed that K uptake and its deposition in tissues by the plant is reduced under salt stress (Lazof and Bernstein, 1999). This reduced potassium concentration in plant tissues grown under salt stress conditions is reported by many authors (Hu and Schmidhalter, 1997; Khan et al., 2000; García et al., 2004; Carter et al., 2005). This decrease is attributed to the antagonistic effects of Na^+^ and K ions (Suhayda et al., 1990).

The present study revealed a significant interactive effect of salt and water stress on Na^+^ and Cl^–^ uptake. Increasing salt stress increased Na^+^ and Cl^–^ uptake. Water stress also increased the Cl^–^ uptake even at low salinity stress. Cl^–^ uptake increased with increasing water stress at the lowest salinity level S1 only. Under the highest salinity level of S4 Cl^–^ uptake decreased with increasing water stress levels.

An important “salt includer,” Jojoba also accumulated significant amounts of sodium under slat stress (Mills and Benzioni, 1992). Na^+^ content in shoots increased sharply across the salt levels in *Atriplex canescens* (Glenn and Brown, 1998) *Salicornia rubra* (Khan et al., 2001) *Bruguiera cylindrica* (Atreya et al., 2009) sea aster (*Aster tripolium* L) (Ueda et al., 2003), jojoba explants (Roussos et al., 2007), *Atriplex halimus* (Martínez et al., 2005), and *S. portulacastrum* (Slama et al., 2008).

Halophytic species *Salicornia rubra* was studied by Khan et al. (2001). Chloride concentration in shoots increased with increasing irrigation water salinity. Another halophyte *Aster tripolium* L was evaluated by Ueda et al. (2003) under water stress and NaCl (300 mM) stress. Cl^–^ content increased three times and Na^+^ content increased up to five times in the NaCl-stressed leaves that of the control. Similarly, *Sesuvium portulacastrum* and *Arthrocnemum macrostachyum* also reported Na^+^ and Cl^–^ compartmentalization (Messedi et al., 2004; Khan et al., 2005).

Under salt stress osmotic adjustment is achieved through increased Na^+^ and Cl^–^ uptake. The production of organic osmotica is more energy-consuming (Greenway and Munns, 1980). Thus inorganic ion accumulation is an alternative mechanism to adjust osmotic potential and seems to save energy, which enables a plant to grow in less favorable conditions (Khalid and Cai, 2011). The shoot acts as a sink for Na^+^ ions when plants were grown under salt stress (Jefferies et al., 1979). Cells are able to avoid high levels of salts in the cytoplasm and achieve osmoregulation by increasing salt levels in the vacuoles by intracellular compartmentalization (Khan et al., 2000, 2005).

### Abscisic acid (μg. g^–1^ FW) and proline content (μg. g^–1^ FW)

Kefu et al. (1991) stated saltbush (*Atriplex spongiosa*) plants did not increase ABA content on 75 mol m^–3^ NaCI salinity but increased at 150 mol m^–3^. Hassine and Lutts (2010) exposed *Atriplex halimus* plants to iso-osmotic stress of NaCl (160 mM) or PEG (15%). Hassine and Lutts (2010) reported that ABA accumulated in response to salt (160 mM NaCl). Alla et al. (2011) while studying *A. halimus* responses for salt (NaCl) or water stress (PEG) found that salt stress produced more metabolic disturbance than water stress. Razzaghi et al. (2011) observed an increase in ABA with increasing water stress.

These ABA-induced stress responses are important for plant survival during both salt and water stress but affect different physiological processes (Hassine et al., 2009). ABA stimulates Na^+^ and Cl^–^ excretion by external salt bladders under salt stress and reduces water loss during water stress (Hassine and Lutts, 2010; Walker and Lutts, 2014). ABA acts as a major signal to regulate transpiration through stomatal pores (Schroeder et al., 2001; Bartels and Sunkar, 2005). ABA-regulated stomatal opening, root growth, and conductance (Schroeder et al., 2001; Sharp and LeNoble, 2002) are important in the avoidance of low water potential. ABA-induced increase of compatible solutes is important for drought avoidance (Ober and Sharp, 1994). The relative root and shoot growth is a response to water stress (Hsiao and Xu, 2000) and is the result of regulation of growth by ABA (Sharp and LeNoble, 2002).

This proline accumulation is involved in osmotic adjustment and protects cellular structures against salt stress and ROS (Hoque et al., 2007). Proline is one of the prominent organic solutes that are stored in the cytoplasm and organelles to balance the osmotic pressure of the ions in the vacuole under stress conditions (Hasegawa et al., 2000). Proline accumulation relates more to osmotic stress than any specific salt effect (Munns, 2002). Proline accumulation is a preventive metabolic adaptation that act as osmoprotectants and antioxidants and/or free radical scavengers (Larher et al., 2009).

Khalid and Cai (2011) reported *M. officinalis* response to proline accumulation by applying various levels of salt and water stress. The highest proline content resulted from combined application of salt and water stress. The same results were reported by Watanabe et al. (2000) and Wu et al. (2015). Teymouri et al. (2009) reported similar results studying three halophytic *salsola* species (*S. rigida*, *S. dendroides*, and *S. richteri*). The maximum increase in proline concentration under salt stress was recorded for *S. richteri*. *Atriplex spongiosa* had a similar trend of decreasing proline content in the range of 50–300 mol/m^3^ but increased rapidly at higher salinities (Storey and Jones, 1979). The same is the case for *Suaeda monoica*, low proline contents were recorded at 500 mol/m^3^ NaCl and below. However, a significant increase was detected at high salinities (Storey and Jones, 1979). Martínez et al. (2005) reported the same results as that of our experiment. He reported that 0 or 15% PEG had no impact on the proline concentration at low NaCl (50 mM) concentration. *Atriplex halimus* showed similar responses after treating seedlings with either NaCl (50, 300, and 550 mM NaCl) or drought (control and withholding water) (Alla et al., 2012). Similar responses of proline to salinity (Bajji et al., 1998) and to osmotic stress (Martinez et al., 2003) had been reported. This can be concluded that proline is efficiently only involved in stress tolerance within the first few hours of stress rather than in long-term stress tolerance (Hassine et al., 2008).

## Conclusion

*Atriplex leucoclada*, showed morphological and physiological adaptations to both salt and water stress and had no negative effect of these stresses on survival percentage. *A. leucoclada* could be classified as obligatory halophyte. *A. leucoclada* resisted maximum salt stress with no significant effect on growth parameters. However, drought stress significantly decreased the growth of *A. leucoclada*. Na^+^ and Cl^–^ content increased not only with increasing salt stress but also with increasing water stress. Cl^–^ uptake in *A. leucoclada* increased with increasing water stress at lowest salinity level S1 only. Both salt and water stress had an additive role in increasing ABA and proline content. Higher salt stress levels increased proline content with increasing water stress levels.

In conclusion, *A. leucoclada* used salt-resistant mechanism to accumulate higher concentrations of salts in the cells. They use physiological adaptation using enzymatic and non-enzymatic mechanisms to cope with the negative impacts of higher salt stress and ROS (Reactive Oxygen Species) produced. *A. leucoclada* could be recommended for production as an alternate for landscape plants and as a fodder crop in areas with salt or brackish water. This will not only save limited available fresh water resources, but also bring more land under cultivation.

## Data availability statement

The raw data supporting the conclusions of this article will be made available by the authors, without undue reservation.

## Author contributions

HA and MZ: conceptualization. MA, NA, TN, AB, SS, and SH: methodology and formal analysis. SF, HFA, and YA: writing – original draft preparation. KL, MH, and SF: writing – review and editing. MZ: supervision. SA, BMA, AM, BA, and NMA: funding acquisition. All authors contributed to the article and approved the submitted version.
